# Purely Off-Clamp Partial Nephrectomy: Robotic Approach Better than Open Using a Pentafecta Outcome with Propensity Score Matching

**DOI:** 10.3390/jcm11216241

**Published:** 2022-10-22

**Authors:** Carlo Gandi, Angelo Totaro, Riccardo Bientinesi, Filippo Marino, Francesco Pierconti, Andrea Russo, Marco Racioppi, Pierfrancesco Bassi, Emilio Sacco

**Affiliations:** 1 Department of Urology, Fondazione Policlinico Universitario A. Gemelli IRCCS—Università Cattolica del Sacro Cuore, 00168 Rome, Italy; 2 Department of Anatomic Pathology and Histology, Fondazione Policlinico Universitario A. Gemelli IRCCS—Università Cattolica del Sacro Cuore, 00168 Rome, Italy; 3 Department of Anesthesiology and Intensive Care, Fondazione Policlinico Universitario A. Gemelli IRCCS—Università Cattolica del Sacro Cuore, 00168 Rome, Italy

**Keywords:** kidney cancer, renal cell carcinoma, partial nephrectomy, RAPN, robot-assisted partial nephrectomy, off-clamp, clampless

## Abstract

Partial nephrectomy (PN) is the gold standard treatment for localized renal masses. Robot-assisted PN (RAPN) has overcome laparoscopy’s technical limitations, greatly expanding the indications of minimally invasive PN, which is dominated by renal artery clamping in almost all published series. We compared off-clamp RAPN (OFFC-RAPN) with the open approach (OFFC-OPN) using propensity score (PS) matching. A favourable pentafecta outcome was defined as a combination of no positive surgical margins (PSM), no complications of Clavien–Dindo (CD) grade ≥ 3, post-operative eGFR loss <10%, length of hospital stay (LOS) ≤ 5 days and estimated blood loss (EBL) < 200 mL. A total of 340 consecutive patients were included. The PS-matched cohort included 142 patients: 71 matched pairs well-balanced for all covariates. The OFFC-RAPN group showed significantly shorter operative time (149.8 vs. 173.9 min, *p* = 0.003), lower EBL (182.1 vs. 329.3 mL, *p* = 0.001), and shorter LOS (5.8 vs. 6.9 days, *p* = 0.02), with a higher proportion of patients with LOS ≤ 5 days (57.7% vs. 23.9%, *p* < 0.001). No significant differences were found for PSM rate (2.8% vs. 8.4%, *p* = 0.27), CD > 2 complication rate (4.2% vs. 2.8%, *p* = 1.00) and mean ± SD eGFR change (−0.06 ± 0.3 vs. −0.8 ± 0.3, *p* = 0.5). Pentafecta was achieved in 56.3% and 21.1% in the OFFC-RAPN and OFFC-OPN series, respectively (*p* < 0.0001). On multivariable analysis, surgical approach and BMI proved to be independent predictors of achieving pentafecta. After adjusting for potential treatment selection bias, OFFC-RAPN outperformed OFFC-OPN for important peri-operative outcomes, without compromising oncological and functional safety.

## 1. Introduction

Partial nephrectomy (PN) is the gold standard treatment for localized renal tumors (cT1-2) [1,2], as it offers oncological outcomes comparable to radical nephrectomy while providing various degrees of preservation of renal function associated with a survival advantage [3,4].

The preservation of renal function after PN seems to be influenced by several factors, most of which are patient-related (e.g., age, preoperative renal function, comorbidities, anatomical complexity of the tumor) and, as such, are unmodifiable [5,6]. On the other hand, the few modifiable factors affecting post-operative renal function are surgeon-dependent, and, apart from the resection [7] and renorrhaphy [8] techniques, the renal ischemia, induced by renal artery clamping, has historically been considered by far the most important [5,9]. An ideal PN includes: negative surgical margins, no postoperative complications and no or minimal impairment of renal function [10]. Thus, transient hilar clamping can provide a bloodless surgical field that facilitates an accurate tumor resection and a precise closure of the renal defect.

However, the introduction of laparoscopic partial nephrectomy (LPN) [11], due to its inherent technical limitations, has brought with it an increasingly unscrupulous use of renal artery clamping in the name of the minimally invasive at all costs. Meanwhile, strong research efforts have been made to determine the optimal warm ischemia time threshold, below which the parenchymal damage cannot be reversible [12,13,14]. In 2010, a publication by Thompson et al. claimed that “every minute counts” when the renal hilum is clamped during partial nephrectomy [9] and, from then on, PN techniques were refined with the aim of shortening the warm ischemia time, not only by shortening the clamping time, but also by using selective clamping or omitting artery clamping.

In this scenario, the advent of robot-assisted PN (RAPN), overcoming laparoscopy’s technical limitations, expanded the indications of minimally invasive PN, bringing the issue of renal artery-clamping back to the center of the debate [15]. Furthermore, some authors identified in RAPN an attractive adaptation to duplicate the performance of OPN, even in more complex cases [16], and, accordingly, LPN should not be considered as a suitable referent for RAPN in comparative studies [17].

Nevertheless, only a few studies have compared RAPN with OPN [18], and no studies have compared the two techniques in a purely off-clamp scenario. At our center, the off-clamp technique has always been the standard approach for partial nephrectomy for both open and robot-assisted surgeries. Therefore, in this study, we aimed to compare the perioperative and early functional outcomes of purely off-clamp RAPN (OFFC-RAPN) with purely off-clamp OPN (OFFC-OPN) using propensity score-matching (PS-matching) analysis to enhance the validity of the comparison [19].

## 2. Materials and Methods

### 2.1. Patient Population, Data Assessment and Surgical Technique

After approval by our local Institutional Review Board (ID: 3876), all consecutive patients undergoing PN at our institution from January 2014 to December 2019 were recorded in a prospective database and included in this study after signing informed consent. Conversion to radical nephrectomy for oncological reasons was an exclusion criterion. Prospectively collected patients’ data were retrospectively analyzed. OFFC-OPN was already a consolidated technique at our center applied to the vast majority of cases, while RAPN was introduced in 2014, starting from the beginning with an off-clamp approach. The surgical technique adopted for RAPN followed a standardized four-arm transperitoneal six-port approach, using a 30° lens, a fenestrated bipolar forceps, monopolar curved scissors and a ProGrasp™ forceps (Intuitive Surgical Inc., Sunnyvale, CA, USA) with da Vinci SI^®^ (Intuitive Surgical Inc., Sunnyvale, CA, USA), from 2014 to December 2015, or da Vinci XI^®^ (Intuitive Surgical Inc., Sunnyvale, CA, USA), from January 2016. In order to avoid bias related to the surgeon’s learning curve, the first 20 consecutive OFFC-RAPN patients were excluded. The majority of OPN and all RAPN procedures included in the present study were performed by the same expert senior surgeon. However, all OPN procedures were performed by three expert senior surgeons.

### 2.2. Measurements and Outcomes

Patients’ demographic and clinical data were obtained from the prospective database. Radiological images were electronically reviewed on our Picture Archiving and Communication Systems database by a senior radiologist with an experienced urologist according to the R.E.N.A.L. nephrometry score [20]. Tumour diameter was evaluated on the axial, coronal and sagittal image planes, and of these the largest diameter was also reported. Furthermore, for the R.E.N.A.L. classification, tumours were stratified into low- (R.E.N.A.L. score 4–6), moderate- (R.E.N.A.L. score 7–9), or high- (R.E.N.A.L. score 10–12) complexity groups.

Operative time (OT) was calculated as the time from skin incision to skin closure. Estimated blood loss (EBL) was recorded in the surgical report, as well as the need for intraoperative blood transfusion. Intra-operative and post-operative complications were stratified according to the Clavien–Dindo classification system [21].

Tumour stage was classified according to the 2009 or 2017 version of the TNM classification [22,23], histological subtypes according to the 2004 version of the WHO classification, and nuclear grade according to the Fuhrman grade. Positive surgical margins were defined as cancer cells at the level of the inked parenchymal excision surface. Estimated GFR (eGFR) was used as a proxy of renal function, and was calculated using the CKD-EPI equation [24]. The baseline eGFR was obtained almost immediately before surgery. For the last eGFR measurement, the serum creatinine nadir during a period of 1–6 months after surgery was used whenever available, and otherwise the nadir during postoperative hospital stay was used. For each patient, chronic kidney disease (CKD) was defined according to the National Kidney Foundation Kidney Disease Outcome Quality Initiative classification [25]. Upstaging of CKD was defined as a deterioration in one class of CKD or more. Percentage eGFR change was calculated as *[(last eGFR—baseline eGFR)/baseline eGFR]*.

Several authors have tried to define the optimal outcome or combination of outcomes after PN [26,27,28], but there is still no universally accepted system for reporting of PN outcomes. Furthermore, all the proposed and widely employed reporting systems are designed for on-clamp partial nephrectomy, as they all include warm ischemia time as a key outcome. In 2018, Brassetti et al. proposed a different trifecta for robot-assisted PN, defined as the coexistence of no positive surgical margins (PSM), no Clavien–Dindo grade ≥ 3 complications and post-operative eGFR loss ≤ 30% [29].

For the aforementioned reasons, in the present study, as primary outcome we introduced a novel “favourable outcome”; that is, a pentafecta defined as a combination of the following endpoints: no PSM, no Clavien–Dindo grade ≥ 3 complications, no post-operative eGFR loss > 10% [28], length of hospital stay (LOS) ≤ 5 days and estimated blood loss (EBL) < 200 mL. LOS cut-off value, as well as EBL cut-off value, were not data-dependent, but identified by the “standard median splits” methodology for categorization of continuous variables [30]. Secondarily, we evaluated independent predictors of the pentafecta outcome (see below).

### 2.3. Propensity Score-Matching Analysis

To balance the preoperative patients’ characteristics, a propensity score-matching analysis was performed. PS-matching is an alternative method for treatment-effect estimation in observational studies by accounting for the conditional probability of treatment selection [19]. PSM involves forming matched groups of treated and untreated subjects who share a similar propensity score. The propensity score is a balancing score defined as the individual probability of treatment assignment conditional on observed baseline covariates. Conditional on the propensity score, the distribution of measured baseline covariates is similar between treated and untreated subjects, thus allowing for reduction of bias when comparing interventions between treatment groups [19]. Continuous and categorical factors were combined to calculate a propensity score for each patient in the study populations using a multivariate logistic regression model based on the following covariates: patients’ age, gender, body mass index (BMI), Charlson comorbidity index (CCI), American Society of Anesthesiologists (ASA) score, solitary kidney, hypertension, diabetes, preoperative estimated glomerular filtration rate (eGFR), preoperative hemoglobin, RENAL score, tumor size and multifocality. The choice of the baseline matched variables was conducted based on previously published series [16,31,32]. Patients in the OFFC-OPN cohort were matched in a 1:1 ratio to patients in the OFFC-RAPN cohort based on the logit of the propensity score, and using a greedy, nearest-neighbour matching algorithm, with a caliper width of 0.285 (20% standard deviations [SDs] of the logit of the propensity score) without replacement [33]. The nearest-neighbour algorithm compares each treated subject with the comparison subject that is closest in terms of the propensity score.

We used numerical and graphical diagnosis to evaluate the common support of the distribution of propensity scores between patients undergoing OFFC-RAPN and those undergoing OFFC-OPN. We compared the multidimensional histograms and kernel density plots of the covariates in the matched OFFC-RAPN and OFFC-OPN groups.

We also performed a sensitivity analysis of the ignorability assumption under PS-matching, which states that all variables simultaneously influencing treatment assignment and outcome have been observed and measured. If there are unobserved factors that influence both treatment assignment and the outcome variables, our estimated effects may be biased (hidden bias). We used Rosenbaum’s bounding approach [34,35] in order to test to what extent our results were sensitive to such unobserved heterogeneity. This approach involves one sensitivity parameter (Γ ≥ 1) that indicates the association (odds) of an unobserved variable with treatment assignment (the higher the value of Γ, the lower the sensitivity of the study to unmeasured confounders).

### 2.4. Statistical Methods

We calculated that the inclusion of at least 80 patients (40 per study group) would allow us to detect a percentage difference for the pentafecta outcome of 30% with a power of 80% and alpha equal to 0.05 [36,37].

To assess the adequacy of the PS-matching process, the standardized mean difference (SMD) in propensity score between matched subjects was calculated, complemented by the comparisons of the baseline covariates and of the cumulative distribution functions of the propensity scores of each matched sample [38]. Both *p* values and SMD were used to compare variables between treatment groups [33]. A SMD of >0.1 (10%) is usually considered to denote meaningful imbalance [39].

Data are presented as mean ± SD or frequency and percentage for continuous and categorical variables, respectively.

The Mann–Whitney U-test was used for comparing differences in continuous outcomes. Particularly, the Mann–Whitney U test is used to compare differences between two independent groups when the dependent variable is either ordinal or continuous, but not normally distributed [40,41]. The Chi-squared or Fisher’s exact test were used to compare proportions.

Predictors of a pentafecta outcome were assessed by stepwise multivariate logistic regression. In both univariate and multivariate analyses, the magnitude of effects was expressed as odds ratios (ORs) with their 95% CIs.

A two-sided *p* < 0.05 was deemed to indicate statistical significance. PS-matching and statistical analyses were performed using MedCalc software for Windows v.12.3.0 (MedCalc Software, Mariakerke, Belgium) and the Statistical Package for the Social Sciences (SPSS), version 21.0 (IBM Corp., Armonk, NY, USA), and Love’s Excel spreadsheet was used for sensitivity analysis (http://www.chrp.org/propensity/ accessed on 1 May 2022).

The study was conducted in compliance with the Strengthening the Reporting of Observational studies in Epidemiology (STROBE) guidelines for reporting observational studies [42], and with the guidelines for reporting propensity score analysis (Supplementary Materials) [43].

## 3. Results

### 3.1. Matching Procedure

**Figure 1:**
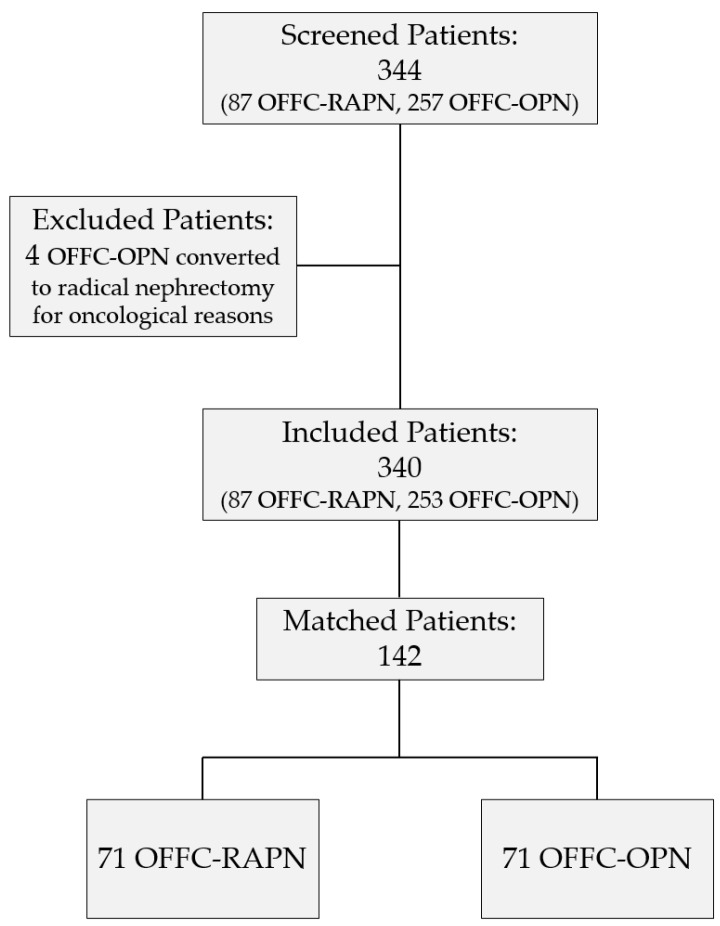
Study flowchart.

Of 340 included patients, 142 (40.2%) were matched according to the propensity score (Figure 1). No data was missing within the prospectively collected database. There were no statistically significant differences between the two cohorts for the variables used for PS-matching (Table 1). Accordingly, a satisfactory degree of overlap in the propensity score between groups was observed, and the SMD in propensity score between matched subjects was not statistically significant (0.004; 95% CI 0.01–0.002; *p* = 0.2). Even for unmatched baseline variables, there were no statistically significant differences between the two matched cohorts (Table 1). Before PS-matching, patients within the RAPN group differed in preoperative tumor characteristics: lower mean RENAL score (4.47 vs. 5.28, SMD −75.02, *p* < 0.0001), lower proportion of tumors with moderate/high RENAL complexity (2.29% vs. 16.19%, SMD −49.44, *p* = 0.003), lower mean tumor size (2.7 vs. 3.59, SMD −44.57, *p* < 0.0001) and lower rate of tumor multifocality (1.14% vs. 7.5%, SMD −31.94, *p* < 0.01). In addition, patients in the RAPN group, before PS-matching, showed a lower mean BMI (25.9 vs. 28.2, SMD −45.66, *p* = 0.001) and suffered less from hypertension (48.27% vs. 61.66%, SMD −26.33, *p* = 0.03).

### 3.2. Primary Outcome

Table 2 shows perioperative and early oncological and functional outcomes after surgery. Within the PS-matched cohort, a significantly higher proportion of patients in the RAPN group reached the pentafecta outcome (56.3% vs. 21.1%, *p <* 0.0001). Furthermore, within the PS-matched cohort, the RAPN group was associated with significantly lower EBL (182.1 vs. 329.3 mL, *p* = 0.001) and shorter LOS (5.8 vs. 6.9 days, *p* = 0.02), with a higher proportion of patients with LOS ≤ 5 days (57.7% vs. 23.9%, *p* <0.001). Operative time was significantly shorter for the RAPN group (149.8 vs. 173.9 min, *p* = 0.003).

With respect to the renal functional outcomes, the mean last eGFR was 83.3 ± 24.7 and 78.2 ± 30.4 mL/min per 1.73 m^2^ (*p* 0.29), with a mean −6 ± 30 and −8 ± 30 percentage change (*p* 0.58) in the RAPN and OPN groups, respectively. A total of 21.1% (15/71) of patients in the RAPN group and 25.3% (18/71) in the OPN group were CKD-upstaged.

No significant differences were found in terms of PSM rate (2.8% vs. 8.4%, *p* = 0.27) between the RAPN and OPN groups in the PS-matched cohort. In both groups, most of the treated tumors were pathologic stage T1 (98.6% vs. 95.8%, *p* = 0.87). pT1b tumors were 5.6% (4/71) and 12.7% (9/71) in the RAPN and OPN groups, respectively (*p =* 0.16).

Regarding peri-operative complications, no significant differences between RAPN and OPN groups were found for both intraoperative (1.4% vs. 7.0%, *p* = 0.21) and postoperative (15.5% vs. 16.9%, *p* = 1.00) complication rates. Likewise, no significant differences between RAPN and OPN groups were found for Clavien–Dindo ≥3 complications (4.2% vs. 2.3%, *p* = 1.00).

### 3.3. Secondary Outcome

In the multivariable analysis, BMI (OR 0.89, 95% CI 0.80–0.99, *p* = 0.04) and surgical approach (RAPN vs. OPN, OR 3.96, 95% CI 1.60–9.79, *p* = 0.002) were independent predictors of obtaining a pentafecta outcome (Table 3), while no significant association was found for age, gender, CCI, ASA, preoperative eGFR, preoperative hemoglobin, R.E.N.A.L. score and tumor size.

### 3.4. Sensitivity Analysis and Diagnostics

Results of the sensitivity analysis for the primary outcome are shown in Table 4. The treatment effect turns insignificant at a critical Γ value of 2.45. This means that an unobserved variable could cause a difference as high as 145% in the odds of receiving RAPN instead of OPN for two subjects with the same baseline characteristics, without changing the inference of our result. In other words, the study is insensitive to a bias that would more than double the odds of assignment to RAPN vs. OPN, instead of no influence by our assumption. We can conclude that the study is reasonably robust to unobserved heterogeneity.

**Figure 2:**
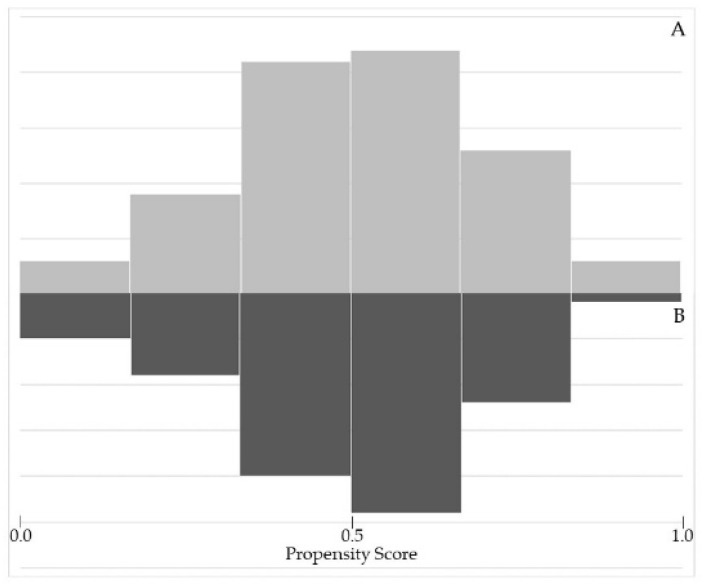
Assessing common support assumption required for propensity score matching procedure (A = RAPN group; B = OPN group).

Figure 2 shows that there is an overlap of the propensity scores of the patients undergoing OFFC-RAPN and those undergoing OFFC-OPN, which clearly shows that the assumption of common support holds in this study.

**Figure 3:**
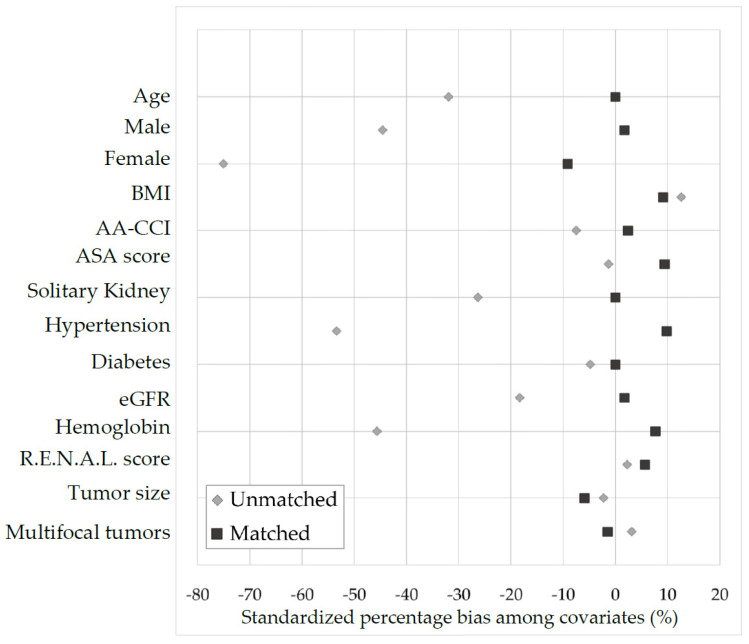
Covariate balance between OPN and RAPN patients using standardized percentage bias across covariates.

The diagnostic graph assessment of the covariate balance (Figure 3) between OFFC-RAPN and OFFC-OPN patients showed that standardized percentage bias among covariates between the two groups reduced drastically after matching.

## 4. Discussion

To our best knowledge, this is the first propensity-score-matched analysis comparing RAPN with OPN in a purely off-clamp scenario. Furthermore, we introduced a novel, widely applicable, pentafecta outcome, taking into account not only oncological, functional and intraoperative surgical outcomes but also post-operative surgical outcomes. In the OFFC-RAPN group, significantly more patients reached the pentafecta outcome. After adjusting for potential treatment selection biases, the robot-assisted approach to off-clamp partial nephrectomy outperformed the open approach in several important perioperative outcomes, including operative time, estimated blood loss and length of hospital stay. Importantly, at multivariable analysis, surgical approach was found to be an independent predictor of achieving the favourable pentafecta outcome.

Only a few of the previous comparative studies testing RAPN vs. OPN reported off-clamp patients, always as a minor part of the study population [18,36,44].

Traditionally, clamping the renal artery during PN is employed to obtain a virtually bloodless resection field, helping the control of surgical margins, although exposing the renal parenchyma to ischemic injury [45]. The optimal warm ischemia time is still a matter of debate. It has been suggested that limited periods of warm ischemia time (<20–25 min) might have a negligible effect on renal function [46]. Nevertheless, when considered as a continuous variable, warm ischemia time is significantly associated with short- and long-term decreased renal function, suggesting that each increasing minute of ischemia carries additional risks for renal function [9]. A lower incidence of postoperative acute kidney injury and CKD after off-clamp PN in solitary kidneys suggested the use of this approach even for patients with solitary tumor and normal contralateral kidney [47]. In a recent PS-matched comparison of long-term functional outcomes, eight years after surgery, patients undergoing off-C PN had a higher probability of maintaining unmodified eGFR (58% vs. 4%, *p* 0.02), and significantly lower probability of experiencing eGFR decrease >25% (9% vs. 47%, *p* 0.02) when compared with patients undergoing on-C PN [31]. Renal ischemia remains the strongest modifiable surgical risk factor for decreased renal function after PN [13]; therefore, efforts should be pursued to avoid ischemic injury during PN.

The introduction of RAPN offered several benefits for urologists: articulating instruments and magnified three-dimensional vision to facilitate precise tumor resection, tumor bed hemostasis and renal reconstruction [48]. Therefore, RAPN may help surgeons achieving the standards of OPN while offering a minimally invasive approach, bridging the gap between LPN and OPN.

In our series, operative time was significantly shorter for RAPN (150 min vs. 174 min, *p* 0.003), in line with the results of the on-clamp series reported by Han et al. (162 min vs. 187 min, *p* < 0.001) [49], while in contrast with several previous studies all based on the on-clamp technique, in which operative time seemed to be a drawback of RAPN [18,50].

In a 2017 meta-analysis, Xia et al. reported that operative time did not differ significantly between the robotic and open approaches after performing a sensitivity analysis of the available data [44]. If RAPN operative time is influenced by the time needed for preparation and docking of the robot [32], the open approach is burdened by lumbotomy, which can be very time-consuming, especially in the phase of closing the abdominal wall.

The advantage of RAPN over OPN in terms of EBL (182 mL vs. 329 mL, *p* 0.001) was confirmed even in our purely off-clamp scenario, in agreement with the results of the most relevant comparative on-clamp studies [18,36]. This finding could be explained by increased abdominal pressure related to the pneumoperitoneum and magnified intra-operative vision, allowing a higher control of bleeding during tumor excision with the robotic approach [51]. Interestingly, mean EBL values of our OFFC-RAPN (182 mL) and OFFC-OPN (329 mL) series, respectively, were within the range of values reported in the previously published predominantly on-clamp studies comparing RAPN (57.5 mL–212 mL) vs. OPN (183 mL–653 mL) [44,52].

In terms of LHS, RAPN confirmed its superiority over OPN even in our off-clamp population (5.8 ± 2.3 d vs. 6.9 ± 3.9 d, *p* 0.02), with 58% of OFFC-RAPN patients being discharged in five or less days after surgery compared to only 24% of the OFFC-OPN group (*p* 0.0001). This finding is in line with the predominantly on-clamp literature [18]. Lee et al. reported that RAPN had favorable outcomes not only in terms of LHS (6.2 vs. 8.9 d, *p* < 0.001) but also of analgesic use (ketoprofen, 0.26 vs. 0.88 ampules, *p* < 0.001) [17]. According to Wang et al., RAPN advantage is maintained even when the comparison involves special population groups, such as patients with highly complex tumors and patients with chronic kidney disease [53]. The shorter LHS associated with RAPN translates into decreased cost of hospitalization that, as highlighted by Laydner et al., could offset the high cost of robotic instrumentation [54].

No differences were found between the OFFC-RAPN and OFFC-OPN groups for both intraoperative (1.4% vs. 7%, *p* 0.23) and postoperative complications (15.5% vs. 16.9%, *p* 1.00). Most complications were Clavien–Dindo grade 2 or less, without statistically significant differences between the robotic and the open approach (11.3% vs. 14.1%, *p* 0.80). Our findings are in agreement with several on-clamp series [32,52,55,56], while based on the results of two meta-analysis, patients undergoing RAPN had a lower rate of postoperative complications when compared to OPN [36,44].

As to oncological safety, in our study a numerically lower PSM rate was noted in the OFFC-RAPN group (2.8% vs. 8.4%, respectively), although not statistically significant (*p* 0.27). The majority of the published studies used PSM as a surrogate proxy of oncological outcomes, even though it cannot truly reflect cancer control. In a meta-analysis involving 1068 patients, Wu et al. concluded that there was no significant difference regarding PSM between RAPN and OPN, but there was a higher recurrence rate in the open group (2.2% vs. 0.4%) [36].

The strengths of our study remain in the purely off-clamp scenario in which RAPN and OPN were compared in the use of the PS-matching methodology, making the groups under comparison uniform in relation to major potential selection biases, and in our compliance with the STROBE guidelines and the guidelines for reporting propensity score analysis, ensuring high-quality reporting of observational studies. PS-matching was applied to balance groups and correct treatment selection bias; however, prediction probabilities of logistic regression may have been biased by the data disbalance between the matched population and the unmatched population in terms of OPN to RAPN ratio (1:1 in the matched population and 2.9:1 in the unmatched population). Limitations of the study also include its limited sample size and retrospective nature, even if based on a prospectively collected database, and the lack of high-complexity RENAL score cases in the matched population as a consequence of the PS-matching process itself. Furthermore, the study may be underpowered with respect to some comparisons, such as complication rate. Finally, oncological equivalence was defined on the basis of the PSM rate as, in our series, a sufficiently long follow-up was not available to evaluate a potential difference in recurrence rate.

In conclusion, in a purely off-clamp scenario, RAPN appears to be superior to OPN in terms of important peri-operative outcomes, without compromising functional and oncological safety. Larger series, possibly with a prospective design, including higher-complexity cases, are needed to confirm our results along with a longer follow-up to investigate the oncological outcomes more deeply.

## Figures and Tables

**Table 1:** Comparison of baseline characteristics between groups in the unmatched and matched populations.

Mean ± SD or *n* (%)	Unmatched Population				Matched Population			
	RAPN	OPN	SMD	*p* Value	RAPN	OPN	SMD	*p* Value
Matched Variables	(*n* = 87)	(*n* = 253)			(*n* = 71)	(*n* = 71)		
Age, years	61.34 ± 12.43	60.93 ± 13.92	0.0311	0.87	60.81 ± 12.99	61.01 ± 14.31	−0.0146	0.600
Gender, *n* (%)				0.29				0.189
Male	52 (59.77)	154 (60.86)	−0.0224		41 (57.74)	43 (60.56)	−0.059	
Female	35 (40.22)	99 (39.13)	0.0225		30 (42.25)	28 (39.43)	0.0569	
BMI, kg/m^2^	25.90 ± 4.05	28.20 ± 5.86	−0.4566	0.001	26.24 ± 4.06	25.90 ± 4.74	0.077	0.472
AA-CCI	4.51 ± 1.55	4.82 ± 1.82	−0.1833	0.12	4.58 ± 1.64	4.55 ± 1.76	0.0176	0.916
ASA score	2.07 ± 0.33	2.09 ± 0.48	−0.0485	0.63	2.07 ± 1.35	2.07 ± 0.52	0,00	0.951
Solitary kidney, *n* (%)	1 (1.14)	15 (5.92)	−0.5341	0.05	1 (1.40)	0	0.0987	0.477
Hypertension, *n* (%)	42 (48.27)	156 (61.66)	−0.2633	**0.03**	36 (50.70)	36 (50.70)	0,00	0.932
Diabetes, *n* (%)	14 (16.09)	42 (16.60)	−0.0135	0.05	13 (18.30)	10 (14.08)	0.0941	0.466
eGFR, mL/min/1.73 m^2^	82.94 ± 20.95	87.79 ± 88.71	−0.0752	0.69	83.10 ± 22.13	82.52 ± 25.29	0.0244	0.794
Hemoglobin, g/dL	13.86 ± 1.32	13.67 ± 1.67	0.1262	0.20	13.75 ± 1.41	13.60 ± 1.84	0.0915	0.401
R.E.N.A.L. score	4.47 ± 0.75	5.68 ± 1.33	−0.7502	**<0.0001**	4.57 ± 0.79	4.65 ± 0.95	−0.0915	0.873
Tumor size, cm	2.70 ± 1.91	3.59 ± 2.08	−0.4457	**<0.0001**	2.90 ± 2.05	2.87 ± 1.31	0.0174	0.597
Multifocal tumors, *n* (%)	1 (1.14)	19 (7.50)	−0.3194	**0.01**	1 (1.40)	1 (1.40)	0,00	0.476
**Unmatched Variables**								
R.E.N.A.L. complexity, *n* (%)				**0.003**				1.000
Low (4–6)	85 (97.70)	212 (83.79)	0.4944		67 (94.35)	66 (92.95)	0.0574	
Moderate (7–9)	2 (2.29)	38 (15.01)	−0.4337		4 (5.65)	5 (7.04)	−0.0574	
High (10–12)	0	3 (1.18)	−0.1545		0	0		
Tumor laterality, *n* (%)				0.118				0.1569
Right	38 (43.67)	137 (54.15)	−0.2108		32 (45.07)	33 (46.47)	−0.0281	
Left	49 (56.32)	116 (45.84)	0.2108		39 (54.92)	38 (53.52)	0.0281	
ECOG score	1.20 ± 0.40	1.28 ± 0.52	−0.1724	0.929	1.22 ± 0.41	1.23 ± 0.46	−0.0229	0.929

BMI = body mass index; AA-CCI = age-adjusted Charlson comorbidity index; eGFR = estimated glomerular filtration rate.

**Table 2:** Results for primary outcome.

Mean ± SD or *n* (%)	Matched Population		
Surgical, Pathological and Functional Outcomes	RAPN	OPN	*p* Value
	*(n* = 71)	*(n* = 71)	
Operative time, min	149.8 ± 41.1	173.9 ± 51.8	**0.003**
EBL, mL	182.1 ± 198.9	329.3 ± 305.6	**0.001**
Tranfusions, *n* (%)	5 (7.0)	4 (5.6)	1.00
LHS, days	5.8 ± 2.3	6.9 ± 3.9	**0.02**
LHS ≤ 5, *n* (%)	41 (57.7)	17 (23.9)	**0.0001**
Complications, *n* (%)			
Intra-operative	1 (1.4)	5 (7.0)	0.21
Post-operative	11 (15.5)	12 (16.9)	1.00
C-D 1–2	8 (11.3)	10 (14.1)	0.8
C-D 3–4	3 (4.2)	2 (2.8)	1.00
PSM, *n* (%)	2 (2.8)	6 (8.4)	0.27
Tumour histology, *n* (%)			
Malignant	48 (67.6)	47 (66.2)	1.00
Clear cell RCC	32 (45.1)	29 (40.8)	0.73
Papillary RCC	13 (18.3)	11 (15.5)	0.82
Chromophobe RCC	2 (2.8)	7 (9.9)	0.17
Others	1 (1.4)	0 (0)	1.00
Benign	23 (32.4)	24 (33.8)	1.00
Angiomyolipoma	8 (11.3)	7 (9.9)	1.00
Oncytoma	13 (18.3)	14 (19.7)	1.00
Others	2 (2.8)	3 (4.2)	1.00
Pathological stage, *n* (%)			
T1a	65 (91.55)	59 (83.09)	0.14
T1b	4 (5.63)	9 (12.68)	0.16
T2a	0 (0)	0 (0)	1.00
T2b	1 (1.41)	2 (2.81)	0.97
T3	1 (1.41)	1 (1.41)	1.00
Fuhrman grade, *n* (%)			
Low (1–2)	40 (56.3)	36 (50.7)	0.12
High (3–4)	3 (4.2)	4(5.6)	1.00
Not specified	5 (7.0)	7 (9.9)	0.76
Last eGFR, mL/min per 1.73 m^2^	83.3 ± 24.7	78.2 ± 30.4	0.29
Change in eGFR	−0.06 ± 0.3	−0.08 ± 0.3	0.58
CKD upstaging, *n* (%)	15 (21.1)	18 (25.3)	0.69
Pentafecta outcome, *n* (%)	40 (56.3)	15 (21.1)	**<0.0001**

EBL = estimated blood loss; LHS = length of hospital stay; C-D = Clavien–Dindo; PSM = positive surgical margin; eGFR = estimated glomerular filtration rate; CKD = chronic kidney disease. Pentafecta outcome = no PSM + no complications CD > 2 + EBL < 200 mL + LHS ≤ 5 + no post-operative eGFR loss >10%.

**Table 3:** Results for secondary outcome: univariate and multivariate logistic regressions for predictors of achieving the pentafecta outcome.

	Unmatched Population				Matched Population			
	(*n* = 340)				(*n* = 142)			
	Univariate Analisys		Multivariate Analysis		Univariate Analisys		Multivariate Analysis	
Predictors of Pentafecta Outcome	OR (95% CI)	*p* Value	OR (95% CI)	*p* Value	OR (95% CI)	*p* Value	OR (95% CI)	*p* Value
Age	0.97 (0.95–1.00)	0.05	0.96 (0.94–0.99)	**0.04**	0.97 (0.94–0.99)	**0.04**	0.96 (0.93–1-00)	0.06
Gender: F vs. M (ref.)	1.12 (0.58–2.15)	0.72	/	/	1.5 (0.70–3.28)	0.28	/	/
BMI	0.89 (0.83–0.96)	**0.003**	0.94 (0.86–1.02)	0.14	0.90 (0.82–0.99)	**0.04**	0.89 (0.80–0.99)	**0.04**
AA-CCI	0.82 (0.67–1.00)	0.05	0.91 (0.72–1.16)	0.47	0.80 (0.63–1.01)	0.05	0.89 (0.68–1.17)	0.42
ASA score	0.79 (0.38–1.64)	0.54	/	/	0.47 (0.18–1.18)	0.11	/	/
Preop. eGFR	0.99 (0.99–1.01)	0.75	/	/	1.00 (0.98–1.02)	0.51	/	/
Preop. Hemoglobin	1.17 (0.95–1.46)	0.14	/	/	1.19 (0.92–1.54)	0.17	/	/
R.E.N.A.L. score	0.71 (0.51–0.97)	**0.03**	0.90 (0.55–1.29)	0.58	0.84 (0.53–1.33)	0.46	0.83 (0.49–1.42)	0.50
Tumor size	0.67 (0.51–0.87)	**0.002**	0.74 (0.55–0.99)	**0.04**	0.77 (0.54–1.10)	0.16	0.80 (0.54–1.17)	0.26
RAPN vs. OPN (ref.)	5.91 (3.02–11.59)	**<0.0001**	4.48 (2.07–9.68)	**0.0001**	3.31 (1.45–7.59)	**0.004**	3.96 (1.60–9.79)	**0.002**

BMI = body mass index; AA-CCI = age-adjusted Charlson comorbidity index; eGFR = estimated glomerular filtration rate.

**Table 4:** Sensitivity analysis for the primary outcome variable.

Gamma Values (Γ)	Rosenbaum’s Upper Bound
	Two-Tail *p* Values
1.00	0.0003
1.50	0.0042
2.00	0.0193
**2.45**	**0.0471**
2.50	0.0512
3.00	0.1002
3.50	0.1634
4.00	0.2364
4.50	0.3153
5.00	0.3966
5.50	0.4781
6.00	0.5578

Γ: odds of differential assignment to treatment due to unobserved factor. In a study free of hidden bias, Γ is equal to 1. With increasing Γ, the upper bound increases, reflecting uncertainty about the test statistics in the presence of unobserved selection bias. Γ and upper bound *p*-value for the desired significance level (*p* < 0.05) are in bold.

## Data Availability

Data related to patients and surgical procedures can be found at the Department of Urology, Fondazione Policlinico Universitario A. Gemelli IRCCS—Università Cattolica del Sacro Cuore, Largo Agostino Gemelli 8, 00168 Rome, Italy. The results of histopathologic exams are available from the Department of Anatomic Pathology and Histology, Fondazione Policlinico Universitario A. Gemelli IRCCS—Università Cattolica del Sacro Cuore, Largo Agostino Gemelli 8, 00168 Rome, Italy.

## Supplementary Materials

The following supporting information can be downloaded at: https://www.mdpi.com/article/10.3390/jcm11216241/s1, File S1: The guidelines for reporting propensity score analysis (modified from the STROBE statement).
