# Supplementary material for: Purely Off-Clamp Partial Nephrectomy: Robotic Approach Better than Open Using a Pentafecta Outcome with Propensity Score Matching
Source: J Clin Med. 2022 Oct 22;11(21):6241. doi: 10.3390/jcm11216241 (PMC9655173; doi:10.3390/jcm11216241)
Supplement: Supplementary file 1 [file jcm-11-06241-s001.zip › jcm-1919213-supplementary.pdf]

File S1: The guidelines for reporting propensity score analysis (modified from the STROBE statement).

Section/topic		Item No	Recommendation
**Title and abstract**	☑	1	Indicate the use of propensity analysis with a commonly used term in the title or the abstract
	☑	2	Provide in the abstract an informative and balanced summary of what was done and what was found
**Introduction**			
Background/rationale	☑	3	Explain the scientific background and rationale for the investigation being reported (cf. para. 1)
Objectives	☑	4	State specific objectives, including any prespecified hypotheses (cf. para. 1)
**Methods**			
Setting	☑	5	Describe the setting, locations, and relevant dates, including periods of recruitment, treatment, follow-up, and data collection (cf. Para. 2.1)
Patient selection	☑	6	Give the eligibility criteria, and the sources and methods of subject ascertainment and selection (cf. para. 2.1)
Variables	☑	7	Clearly define all outcomes, treatments, predictors. Give diagnostic criteria, if applicable (cf. para. 2.2)
Data sources/ measurement	☑	8	For each variable of interest, give sources of data and details of methods of assessment (measurement)(cf. para. 2.2)
Bias	☑	9	Describe how propensity score analysis was used to address bias (cf. para. 2.3)
	☑	10	Describe any other methods to address potential sources of bias, e.g. sensitivity analysis (cf. para. 2.3)
Sample size	☑	11	Explain how the study size was arrived at (cf. para. 2.4)
Statistical analyses	☑	12	Describe all the analytic methods, including the propensity score methods, e.g. matching, weighting, stratification, or covariate adjustment using propensity score (cf. para. 2.3)
	☑	13	Indicate the model used to estimate propensity score, e.g. logistic model, boosting (meta-classifiers), decision trees (cf. para. 2.3)
	☑	14	State the variables included in the propensity score model (cf. para. 2.3)
	☑	15	Explain the variable selection procedure for propensity score model (cf. para. 2.3)
		16	For propensity score matching:
	☑	16.1	Explicitly state the matching algorithm and distance metric (cf. para. 2.3)
	☑	16.2	Indicate matching ratio (1:m matching) (cf. para. 2.3)
	☑	16.3	Indicate whether sampling with or without replacement was used (cf. para. 2.3)
	☑	16.4	Describe the statistical methods for the analysis of matched data (cf. para. 2.4)

	☑	16.5	Describe methods for assessing the comparability of baseline characteristics in the matched groups (cf. para. 2.3)
	☑	17	For propensity score weighting, describe methods for assessing the comparability of baseline characteristics in the weighted groups (cf. para 2.3)
		18	For propensity score stratification:
	☐	18.1	Give the number of strata (not applicable)
	☐	18.2	Describe methods for assessing the comparability of baseline characteristics in each stratum (not applicable)
	☑	19	Explain how assumption of propensity score analysis was examined (cf. para. 2.3)
	☑	20	Explain how missing data were addressed, including missing data in propensity score estimation (cf. para. 2.2)
	☐	21	If applicable, describe any methods used to examine subgroups and interactions (not applicable)
	☑	22	Describe any sensitivity analyses (cf. para. 2.3)
	☑	23	Indicate the software used for analysis (cf. para. 2.4)
	☑	24	If applicable, report the package used to create matched sample, e.g. GMATCH macro in SAS, MatchIt package®, Optmatch package ® (cf. para. 2.4)
**Results**			
Participants		25	Report numbers of participants at each stage of study:
	☑	25.1	sample size of patients potentially eligible (cf. para. 3.1)
	☑	25.2	sample size of patients confirmed eligible and included (cf. para. 3.1)
	☑	25.3	sample size of patients analyzed (cf. para. 3.1)
	☑	25.4	for propensity score matching, sample size for each treatment group before and after matching (cf. para. 3.1)
	☑	26	Explain reasons for exclusion at each stage (cf. para. 3.1)
	☑	27	Consider use of a flow diagram (cf. para. 3.1)
Patient characteristics	☑	28	Describe the distribution of baseline characteristics for each group before propensity score analysis (cf. para. 3.1)
		29	For propensity score matching, weighting, or stratification:
	☑	29.1	Describe the distribution of baseline characteristics in the matched/weighted groups or in each stratum (cf. para. 3.1)
	☑	29.2	Describe the results of the comparability of baseline characteristics, whether there are still systematic differences between treatment groups (cf. para. 3.1)
	☑	30	Indicate number of patients with missing data for each variable of interest, especially the variables used in propensity score model (cf. para. 3.1)
Outcome data	☑	31	Report outcomes of each treatment group (cf. para. 3.2 and 3.3)
Main results	☑	32	Give propensity score analysis estimates and their precision, e.g. 95% confidence interval (cf. para. 3.4)

	☑	33	If applicable, give unadjusted estimates and/or adjusted estimates and their precision, e.g. 95% confidence interval. Make clear which additional factors were adjusted for (cf. para. 2.3)
Other analyses	☑	34	Report other analyses done, e.g. analyses of subgroups and interactions, and sensitivity analyses (cf. para. 3.3 and 3.4)
**Discussion**			
Key results	☑	35	Summarize key results with reference to study objectives (cf. para. 4)
Limitations	☑	36	Discuss limitations of the study, taking into account sources of potential bias or imprecision (cf. para. 4)
	☑	37	Discuss both direction and magnitude of any potential bias (cf. para. 4)
Interpretation	☐	38	Discuss whether imbalance of baseline characteristics still exists, and give a cautious interpretation (not applicable)
	☑	39	Give a cautious overall interpretation of results considering objectives, limitations, multiplicity of analyses, results from similar studies, and other relevant evidence (cf. para. 4)
Generalizability	☐	40	For propensity score matching, discuss the possibility and potential influence of incomplete matching, especially the studies in which the matched sample size is less than 50% (not applicable)
**Other information**			
Funding	☑	41	Give the source of funding and the role of the funders for the present study and, if applicable, for the original study on which the present article is based (cf. funding section)

* von Elm E, Altman DG, Egger M, et al. The Strengthening the Reporting of Observational Studies in Epidemiology (STROBE) statement: guidelines for reporting observational studies. J Clin Epidemiol 2008;61(4):344-9.

This guideline can be downloaded at:

https://sites.duke.edu/xiaofeiwang/files/2016/12/Supplementary-Table-6.pdf
